# A Common Concern With a Rare Diagnosis: Pancreatic Neuroendocrine Neoplasms

**DOI:** 10.7759/cureus.17767

**Published:** 2021-09-06

**Authors:** Ecler Jaqua, Van Nguyen, Vincent Pan, Carlos Cereser

**Affiliations:** 1 Family Medicine, Loma Linda University Medical Center, Loma Linda, USA; 2 Surgical Oncology, Santa Casa de Misericórdia de Porto Alegre, Porto Alegre, BRA

**Keywords:** endocrine oncology, pancreas preserving resection, gastrointestinal oncology, pancreas surgery, pancreatic neuroendocrine neoplasms

## Abstract

Pancreatic neuroendocrine neoplasms (pNENs) are rare, representing only a small percentage of all pancreatic tumors. We report the clinical and radiological features of pNENs. Intraoperative pathology confirmed pNENs with clear margins and the patient did not require adjuvant chemoradiation. The patient is currently doing well and being closely monitored due to the high risk of relapse.

## Introduction

Many patients may present to the outpatient, primary care office with abdominal pain, but its etiology is not always obvious. Abdominal pain is associated with increased morbidity whereas high body mass index, tobacco and alcohol use are not [1]. Abdominal pain even accounts for approximately 7%-10% of all emergency department concerns [2]. Although a common concern, potentially life-threatening disorders, such as neoplasm, must be excluded in the evaluation of abdominal pain. Pancreatic neuroendocrine neoplasms (pNENs), previously known as islet cell tumor, are relatively rare representing only 1%-2% of all pancreatic tumors, but has had increased prevalence in recent years due to incidental radiologic findings and physician awareness [3,4].

## Case presentation

**Figure 1:**
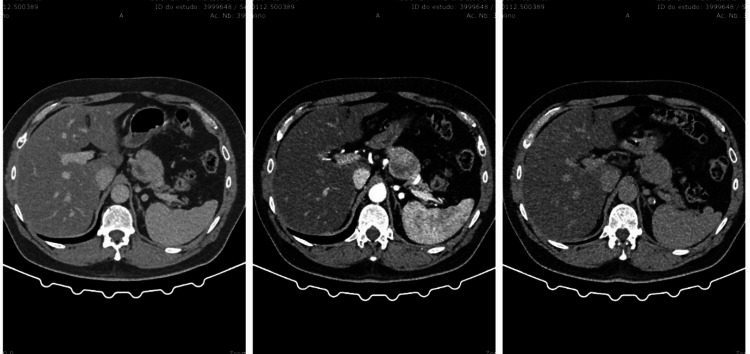
Multiple phase CT scan abdomen and pelvis with contrast (portal-venous, early-arterial, late-arterial phase). Lesion noted to the body of the pancreas.

**Figure 2:**
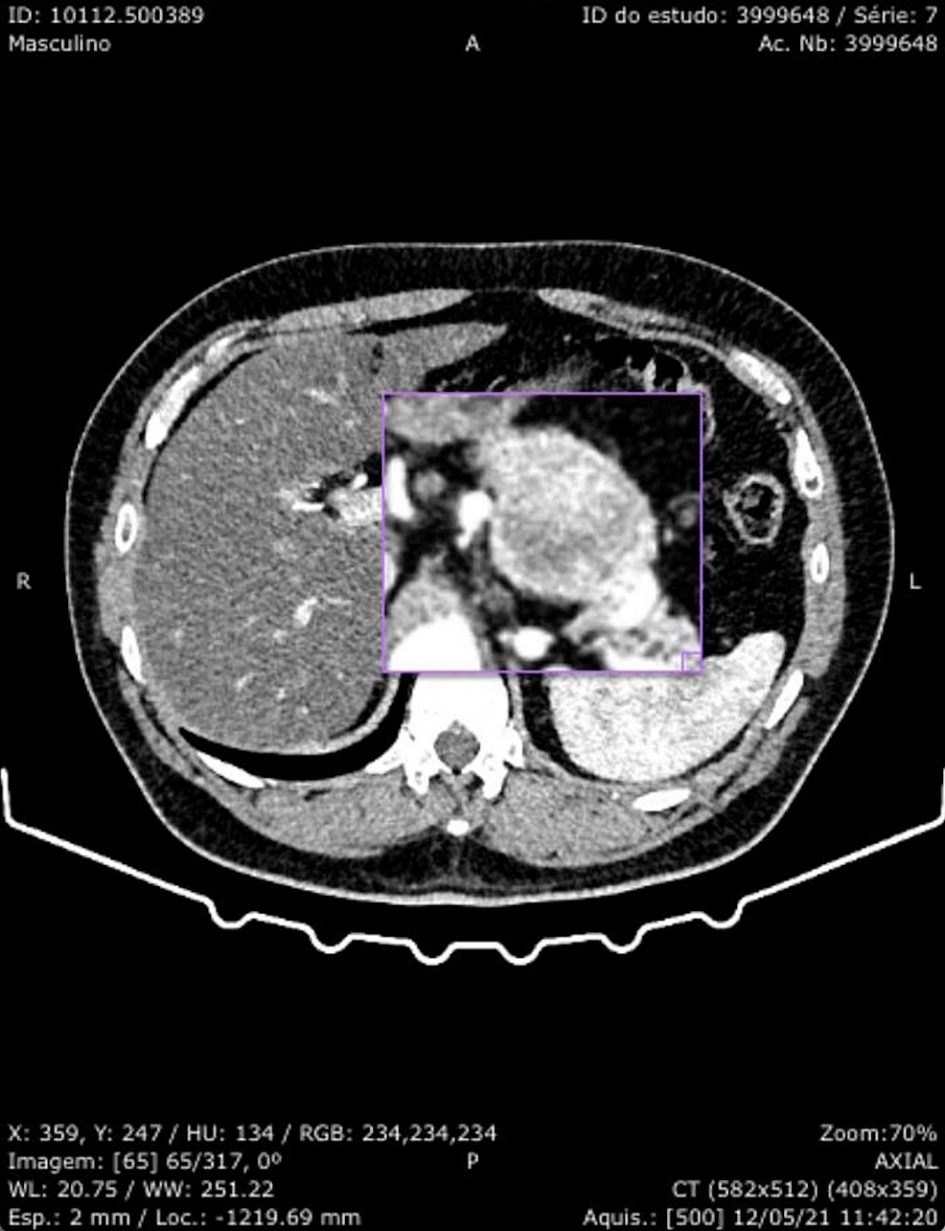
CT scan abdomen and pelvis with contrast; lesion enhanced. Lesion noted to the body of the pancreas.

A 63-year-old male with a history of hypertension, atrial fibrillation, obesity, and gastroesophageal reflux disease, presented with a one-week history of dull, left lower quadrant pain that radiated to the mid-epigastric region, not relieved by acetaminophen. The patient denied any associated weight changes, nausea, blood in stool, changes in bowel patterns, fever, or chills. Physical exam showed an obese abdomen with normoactive bowel sounds and tenderness to palpation diffusely across the abdomen. Due to the acuity of the pain, the patient was started on a proton-pump inhibitor and amoxicillin-clavulanic acid as empiric treatment for dyspepsia and diverticulitis. A computed tomography (CT) of the abdomen and pelvis was also ordered but did not reveal any evidence of diverticulitis. Imaging did not show any lymphadenopathy but did show a 4.6 cm nodular lesion in the body of the pancreas, suggestive of a neoplastic process, and a second 1.1 cm cystic lesion in the head of the pancreas (Figures 1-2).

These imaging findings were discussed with the patient and urgent laboratory work and further diagnostic imaging were ordered. Labs, including complete blood count, metabolic panel, amylase, lipase, blood sugars, carcinoembryonic antigen (CEA), and carbohydrate antigen (CA) 19-9 were unremarkable. Positron emission tomography (PET) confirmed increased metabolic activity in the body of the pancreas, measuring 3.7 x 3.5 cm.

**Figure 3:**
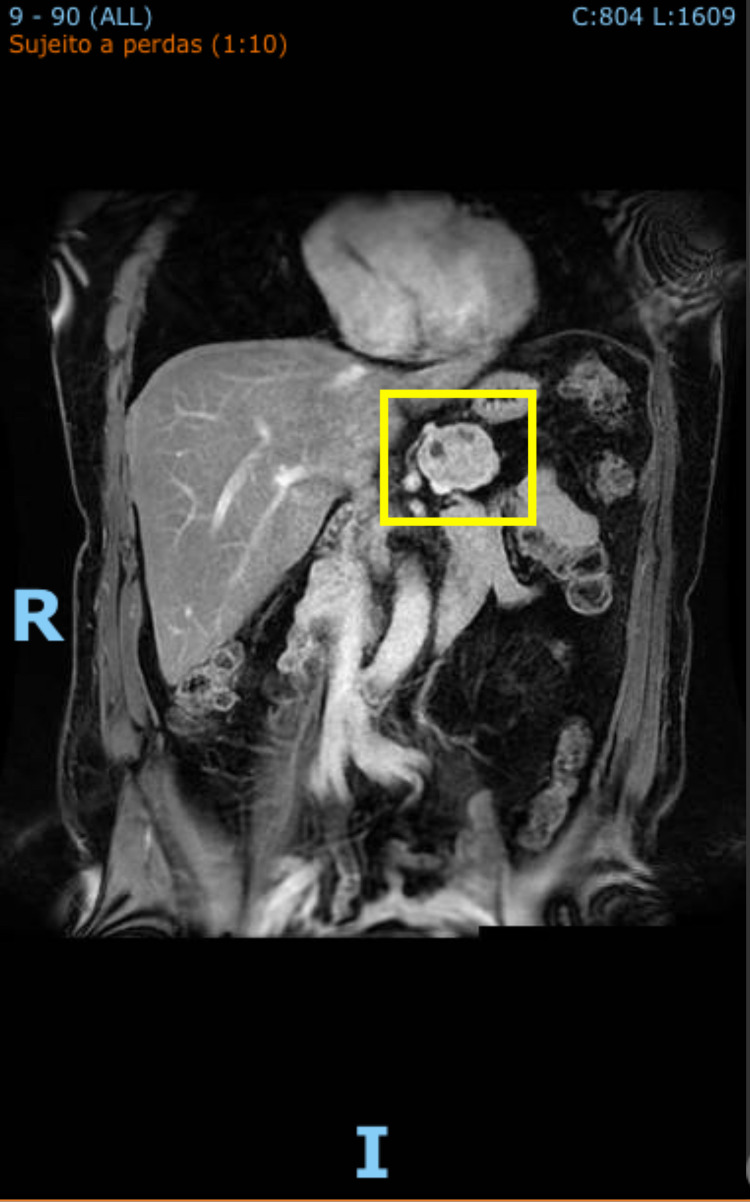
MRI abdomen and pelvis with contrast; lesion marked. Lesion noted to the body of the pancreas.

Although increased metabolic activity was isolated to the pancreas, the patient underwent magnetic resonance imaging (MRI) to further evaluate the liver for metabolic disease. MRI was more suggestive of neuroendocrine neoplasm without obvious metastasis, but pathology would need to be obtained prior to diagnosis (Figure 3).

**Figure 4:**
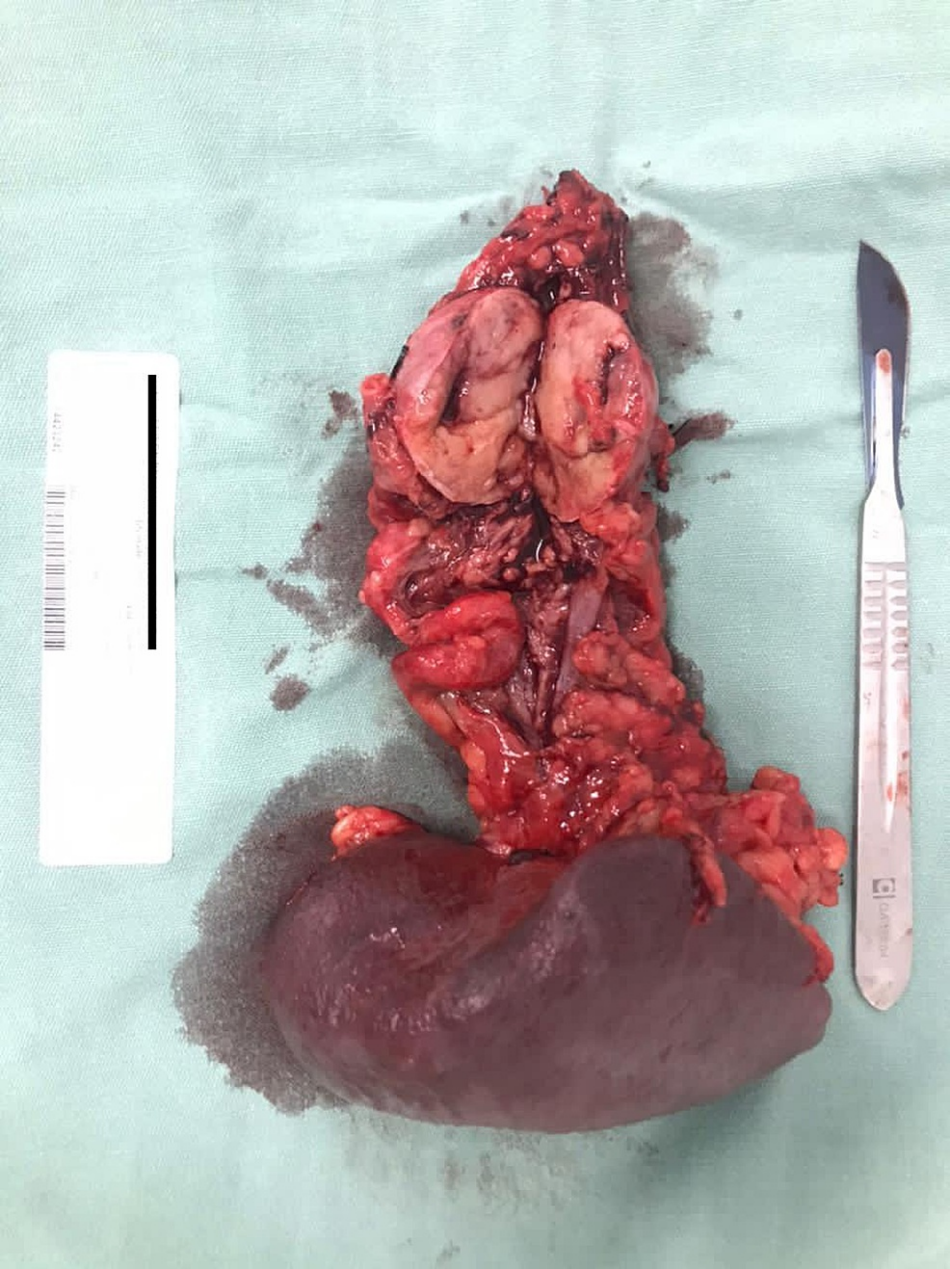
Surgical resection of neoplasm – partial pancreatectomy with total splenectomy.

**Figure 5:**
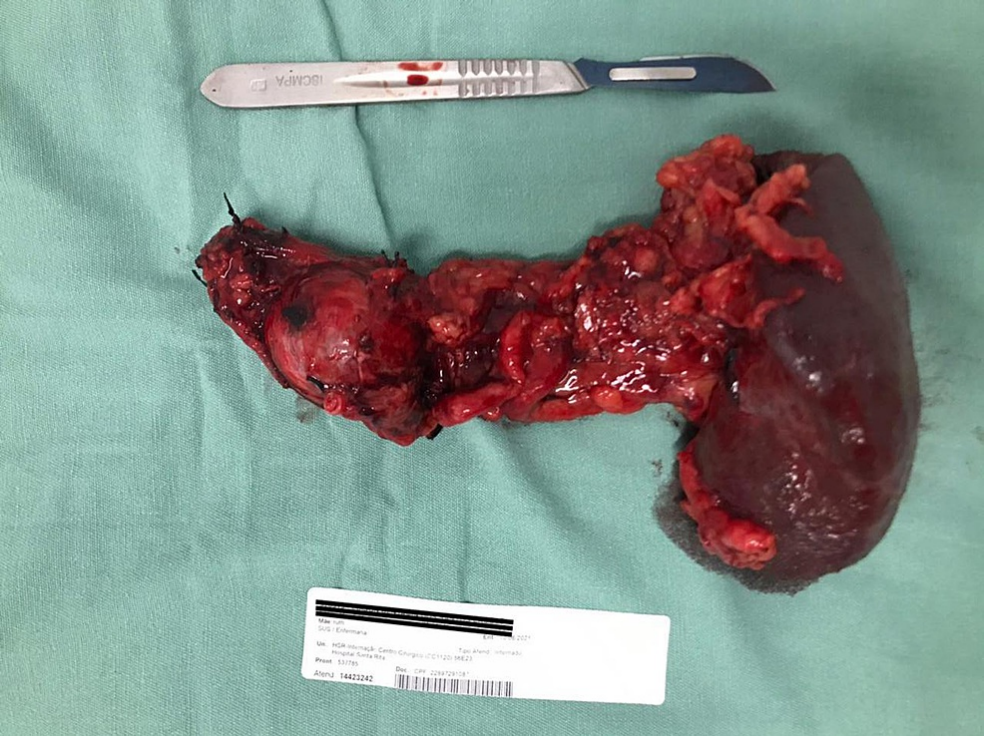
Surgical resection of neoplasm – partial pancreatectomy with total splenectomy.

Approximately four weeks after presentation, the patient underwent an exploratory laparotomy, partial pancreatectomy, and total splenectomy. Pathology confirmed pancreatic neuroendocrine tumor with clear margins (Figures 4-5). The patient had a non-complicated post-operative course with normal blood sugars and was discharged home on postoperative day seven.

## Discussion

The differentiation between functional gastrointestinal disorders and organic disease is difficult. Abdominal pain presenting with unintentional weight loss is associated with organic disease [5]. It must be acknowledged, however, that labeling abdominal pain as “chronic” usually limits the improvement of symptoms and hinders further investigation by clinicians. When used appropriately, advanced imaging, such as CT, can be ordered to rule out more sinister diagnoses such as malignancy. It is due to this imaging, usually requested to evaluate for other diagnoses, that rare neoplasms such as pNENs are increasing in incidence [3,4].

Presentation

Since pNENs can be functional or non-functional, the clinical presentation can vary depending on their type. The most common functional pNENs are insulinoma (40%-55%), gastrinoma (25%-50%), glucagonoma, and VIPoma [6]. Non-functional pNENs are asymptomatic and usually incidental findings. Symptoms of non-functional pNENs are due to tumor burden - abdominal pain (35%-78%), anorexia and nausea (45%), and weight loss (22%-35%) [7]. The majority of pNENs are sporadic cases but a small minority are associated with hereditary diseases such as multiple endocrine neoplasia type 1, von Hippel Lindau, neurofibromatosis type 1, and tuberous sclerosis [8]. pNENs tend to have multifocal metastases. The most common site of metastases is to the liver, lungs, bone, peritoneum, adrenal glands, and spleen [9].

Diagnosis

Due to its wide availability and high sensitivity and specificity, CT has become the diagnostic imaging of choice for pNENs. Helical (spiral), multiphasic, or contrast-enhanced CT should be used as pNENs are highly vascular. MRI may be a favorable test to characterize smaller pancreatic lesions and metastasis to the liver [10]. PET can be used to further localize neoplasms [11]. The last modality that can be used, endoscopic ultrasonography (EUS), can be used to provide high-resolution imaging of the pancreas, with higher sensitivity than CT. The other benefit of EUS is that fine-needle aspiration can be done at the same time as imaging [12].

Treatment

Small pNENs, less than 2 cm, can be followed with observation. Surgical resection is still the mainstay of treatment, a potentially curative option for pNENs [13]. Localization of pNENs, as well as the grade and biological activity of the tumor, lymph node involvement, metastasis, surgical margins, tumor size, and macro/microvascular involvement, affect surgical cure rate and overall prognosis. Due to the high relapse rate of pNENs, adjuvant systemic therapy such as chemotherapy, somatostatin analogs, targeted inhibitor therapies, and peptide receptor radionuclide therapy should be considered.

Prognosis

Resection of localized pNENs has a five-year survival rate of 55%. Non-resectable pNENs have a five-year survival rate of 15% [14]. TNM system (tumor, node and metastasis) is the most common and recognized cancer staging system. pNENs can also be staged by their grade (G) and degree of differentiation going from G1 to G3. G1-grade tumors, in comparison to G3-grade tumors, have a median survival of twelve years and ten months, respectively [14]. The higher the tumor grade is, the more poorly differentiated cells are and consequently worsen prognosis.

## Conclusions

Patients with abdominal pain and perhaps unexplained weight loss should be suspected of malignancy and undergo diagnostic imaging via the widely available, and highly sensitive and specific CT. In the case of localized pNENs, surgical treatment can be curative but may still require adjuvant therapies due to its high frequency of relapse.
